# Probability Distribution Characteristics of Horizontal and Vertical Mechanical Properties of Rubber Bearings

**DOI:** 10.3390/ma15228031

**Published:** 2022-11-14

**Authors:** Di Wu, Caiming Li, Zhenyu Yang, Yang Liu, Yan Xiong, Guoping Jiang

**Affiliations:** 1 Earthquake Engineering Research & Test Center, Guangzhou University, Guangzhou 510006, China; 2 Guangdong Provincial Key Laboratory of Earthquake Engineering and Applied Technology, Guangzhou University, Guangzhou 510006, China; 3 Key Laboratory of Earthquake Resistance, Earthquake Mitigation and Structural Safety, Ministry of Education, Guangzhou 510006, China; 4 Fourth Construction Co. of China Construction Eighth Engineering Division Co., Qingdao 266071, China; 5 School of Civil Engineering, Guangzhou University, Guangzhou 510006, China; 6 State Key Laboratory of Subtropical Building Science, South China University of Technology, Guangzhou 510640, China; 7 School of Civil Engineering, Fujian Jiangxia University, Fuzhou 350108, China

**Keywords:** rubber bearing, mechanical property, probabilistic distribution, seismic isolation, horizontal stiffness, vertical stiffness

## Abstract

Rubber bearings are widely used to protect civil structures from destructive earthquakes. The mechanical properties of the bearings are the key technical parameters that determine the seismic isolation performance of isolated structures. To estimate the probability distribution of the mechanical properties related to rubber bearings (including horizontal stiffness, vertical stiffness, post-yield stiffness and yield force) under seismic events. Typical natural rubber bearings (NRBs) and lead-core rubber bearings (LRBs) were designed and fabricated, and the bearings were subjected to repeated load tests using a compression-shear testing machine. The test results of the horizontal and vertical mechanical properties of the bearings in the tests were basically consistent with the design values, and the rubber bearings showed stable mechanical behavior under repeated cyclic loading. The statistical analysis of the test results revealed that the relevant mechanical properties of the NRB and LRB specimens followed a lognormal or general extreme distribution with coefficients of variation mainly ranging from 0.86% to 5.6%. The dispersion of the yield force of LRB was the largest in the repeated tests of many mechanical parameters of typical rubber bearings.

## 1. Introduction

The steel laminated rubber bearing is well recognized as an effective device to protect buildings and bridges from destructive earthquakes [1]. Recently, the rubber bearing, including the thick one, has been adopted in many civil structures, including buildings [2,3], bridges [4] and industrial facilities, and the bearing with vertical isolation is also developed [5]. Although the design of an isolated structure is generally based on deterministic parameters, the seismic behavior of a real-world structure exhibits uncertainties, including the seismic inputs, the dimension of components and the mechanical properties of materials [6]. Further, the uncertainties can significantly affect the responses of structures under random excitations, so the uncertainties are introduced in the seismic analysis of civil structures [7,8,9].

As the primary energy dissipation device, the lead-core rubber bearing shows uncertainties in its actual mechanical properties during the long-term service life [10,11]. Therefore, the influence of uncertainties in rubber bearings on the seismic performance of buildings retrofitted by isolators needs attentions [12]. Based on an isolated frame, it is found that uncertainties in seismic inputs, as well as those in isolators, both has nonnegligible effects on the seismic responses [13]. Besides, the uncertainty in the superstructure also affects the optimal design of the isolator [14]. Particularly, it is pointed out that uncertainties in isolators due to the variation in the temperature result in a ±10% changes in the reliability [15]. The yield force, among various mechanical properties of an isolator, has the most significant influence on the shear force and displacement response of an isolated storage tank [16]. In addition, the acceleration response of a building is significantly affected by the stiffness of isolators [17]. Therefore, to achieve the target reliability, the isolator should meet a required system capacity and a systematic probabilistic procedure is proposed [18].

The mechanical behaviors of a rubber bearing can be described using a mathematical model [18] or several key parameters, like the yield force and stiffness. To estimate the real seismic performance of an isolated structure, uncertainties of the mechanical properties of the isolator is included in the seismic analysis [16]. Generally, the mechanical properties of the isolator are assumed to follow a normal [19] or uniform distribution [20]. For example, the stiffness and yield force of the isolator follow a uniform distribution within 5%, while the properties superstructure follow the normal and lognormal distribution [12]. In another analysis, the mechanical properties of isolators follow a normal distribution with 25% deviation [17]. Further, a covariance within 10% of the initial and post stiffness leads to a 95% safety [21]. However, due to the lack in data, many researchers have to assume the uncertainty distribution of the isolators, which may result in inaccurate results in the probabilistic analysis.

The probabilistic distribution of the mechanical properties of isolators is the key parameter in the reliability estimation of an isolated structure. Moreover, the rubber is a hyper elastic material with complex mechanical properties [22,23]. Therefore, 224 bearings are tested, and the equivalent shear stiffness follows the I-type minimum-value distribution [24]. Based on 38 rubber bearing with various scales, the ultimate horizontal deformation of isolators is found to follow the normal distribution [25]. To improve the accuracy on the mechanical properties, more mechanical tests, which can lower the annual frequency of exceedance, is proposed [26]. Particularly, based on the sensitivity analysis, the variation in the diameter of the lead core significantly affect the mechanical behavior of the lead rubber bearing (LRB) [27]. Further, as there are many factors contributing to the uncertainty of the isolator, the uncertainty in engineering is divided into aleatory and epistemic ones [28,29].

Strong ground shaking causes many times of periodic hysteretic deformation of the isolator. Also, strong earthquakes are always accompanied by multiple aftershocks [30]. Therefore, the uncertainties in the structures should be considered in the mainshock-aftershock sequence [31]. For example, the stiffness and yield force are assumed to follow a uniform distribution under repeated seismic events [12]. However, current researches on the uncertainty of isolators are mainly based on many specimens from various manufacturers. As an isolator may exhibit different mechanical behaviors under such repeated seismic events, the uncertainty of mechanical properties of an isolator subject to repeated external loads need to be investigated. In this study, two types of typical rubber bearings (natural rubber bearings (NRBs) and lead core rubber bearings (LRBs) are tested under different repeated loads. The rubber bearings are tested under incremental and cyclic loads using a compression-shear tester. Then, the mechanical performance characteristics parameters such as geometry, yield force, horizontal and vertical stiffness of the rubber bearing are collected and analyzed. Finally, the uncertainty distribution of the mechanical properties of isolators is derived.

## 2. Materials and Methods

### 2.1. Test Specimen

**Figure 1:**
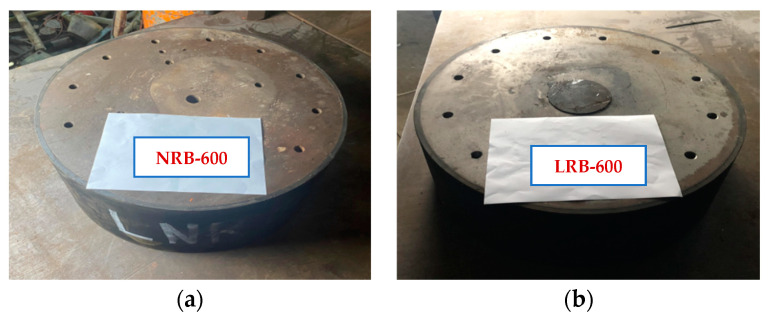
Test specimen of the two rubber bearings: (**a**) NRB-600 specimen; (**b**) LRB-600 specimen.

To obtain the uncertainty of steel-laminated rubber bearings, a natural rubber bearing (NRB-600) and a lead-core one (LRB-600) with a diameter of 600 mm are tested. To study the mechanical properties of rubber bearings under repeated external forces, the test specimens were subject to several mechanical tests. The NRB-600, as shown in Figure 1, is composed of the end plates, several rubber layers and inter-layer steel plates. In addition, the LRB-600 has an additional lead core at the center.

**Figure 2:**
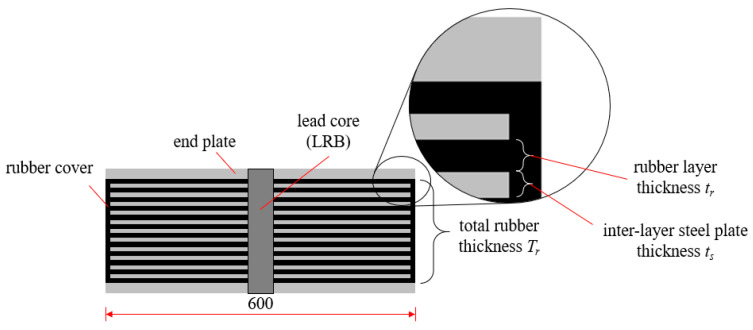
Profile of the specimen (unit: mm).

Table 1 lists the design mechanical and geometric properties of the NRB-600 and LRB-600, while Figure 2 shows the components and definition of the variables. The primarily mechanical properties of the NRB are the vertical stiffness *K_v_* and horizontal stiffness *K_h_*, while the primary properties of the LRB include the vertical stiffness *K_v_*, the horizontal post-yield stiffness *K_d_* and the yield force *Q_d_*. The rubber bearing consists of many layers of steel and rubber layers, the thickness of each steel and rubber layer is *t_s_* and *t_r_*, respectively. The rubber material has a shear modulus *G_s_* of 0.392 MPa, and the total thickness of rubber layers is *T_r_*. According to the test information provided by the manufacturer (Fengze Corporation, Hengshui, China), the yield strength of the lead material, which determine the yield force, is 10.5 MPa, the modulus of elasticity is 16.5 GPa, and the Poisson’s ratio is 0.45. Theoretically, the shear modulus *G_s_*, the total thickness *T_r_* and the diameter D determine the horizontal stiffness of rubber bearings, while *G_s_*, the thickness t_r_ and the diameter D determine the vertical stiffness.

### 2.2. Test Facility and Method

There are several sources of uncertainties in the mechanical properties of the rubber bearings. Typically, the variation in the geometry, material properties and the manufacture procedure all contribute to the uncertainty of a rubber bearing. Meanwhile, an isolator already installed on a building still has uncertainties under repeated loads. Therefore, the specimen was tested for several times to avoid the uncertainty introduced by other factors.

**Figure 3:**
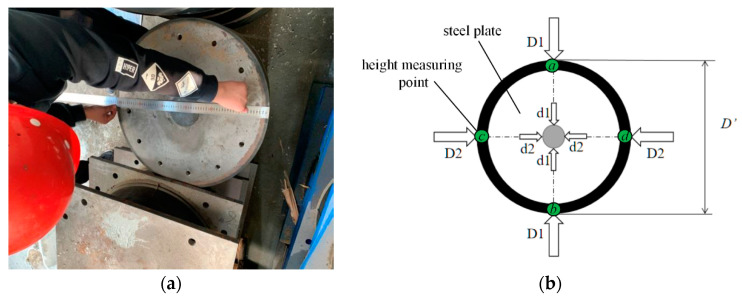
Measure of the specimens before the test: (**a**) photo; (**b**) measure points.

The geometry of the specimen was first measured. As shown in Figure 3, the outer diameter (*D*_1_ and *D*_2_) and inner diameter (*d*_1_ and *d*_2_) were measured. In addition, the height of the specimen was measured at four points (*a*, *b*, *c* and *d*). Table 2 lists the obtained data of the two specimens, and the measured geometry approximates to the design value.

**Figure 4:**
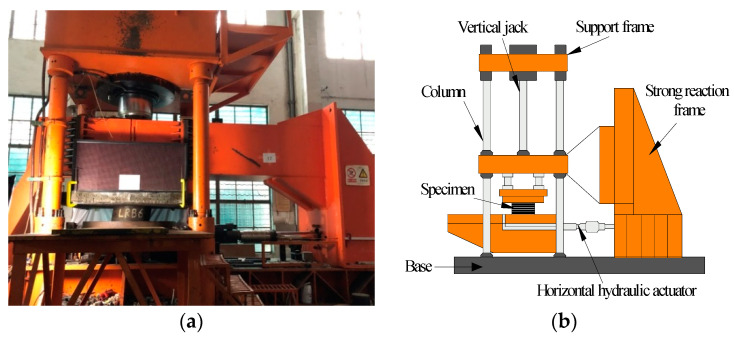
Compression-shear loading testing machine: (**a**) photo; (**b**) components.

The specimens were loaded using a compressive-shear tester, whose vertical and horizontal loading capacity are 20,000 kN ad 2000 kN, respectively, as shown in Figure 4. The specimen was loaded at a certain vertical load, and was subject to a specified horizontal displacement. The allowable horizontal motion of the tester was 500 mm, which is sufficient for the test of a 600 mm diameter specimen.

The specimens were tested according to the International Code Elastomeric seismic-protection isolators-Part 1: Test methods (ISO 22762-1) [32]. The specimens were loaded for three kinds of tests: the incremental and cyclic test, as listed in Table 3. The loading frequency of the test is 0.02 Hz.

The specimens were first subject to incremental loads to obtain the actual mechanical properties using the method proposed in the code. In the incremental test, the specimens were loaded under one-cycle loads with three increasing amplitudes. According to the code, in the horizontal direction, the specimen was subject to a deformation of 50%, 100% and 150% γ_0_, where γ_0_ represents the deformation when the rubber reached 100% shear strain, as specified in the code. In the vertical direction, the specimen was first subjected to a standard vertical load (12 MPa pressure, generated by the gravity of the superstructure), then the vertical load varied for ±30% for three cycles, so the vertical pressure varied between 8.4~15.6 Mpa (a vertical load of 2352~4410 kN, represents the variation in the vertical load during earthquakes).

Then the specimens were subject to cyclic loads to estimate the probabilistic distribution of mechanical properties. In the cyclic test, the specimen was loaded vertically and horizontally for 30 cycles, respectively, to get more data of the mechanical properties under repeated loading. Besides, the specimen was released for 10 min after a scenario was completed. Note that the 30 cycles of the loading procedure had the same amplitude and duration.

The reaction force and the corresponding deformation of the specimen was the key data in the test. The horizontal displacement of the specimen is measured by a built-in senor installed in the tester.

**Figure 5:**
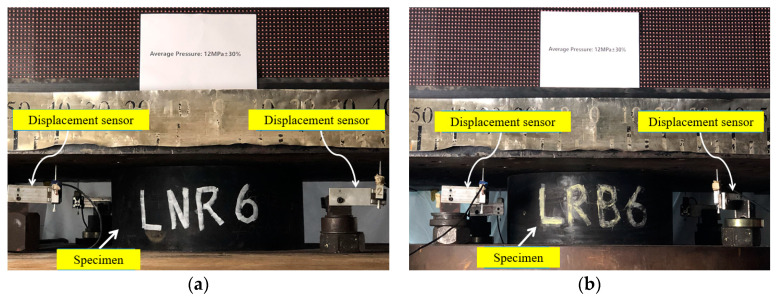
Two specimens (under compression loads) and sensors on the tester: (**a**) NRB-600; (**b**) LRB-600.

Since the vertical deformation of the specimen was quite small, to ensure that the vertical load was uniformly loaded on the support, we installed four displacement transducers to measure the vertical deformation, as shown in Figure 5. The vertical deformation of the specimen is the average value of the four displacement responses monitored by the sensors.

## 3. Results

### 3.1. Results in the Incremental Tests

**Figure 6:**
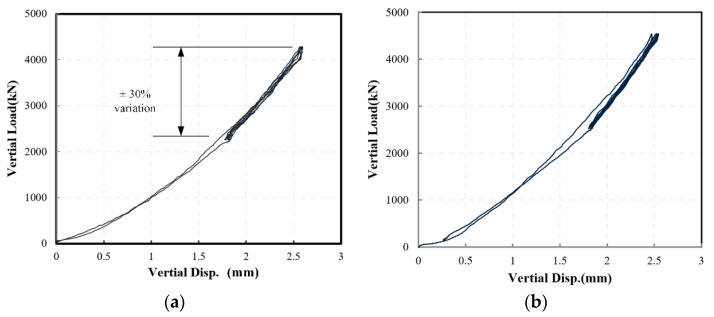
The force-deformation curve of (**a**) NRB-600 and (**b**) LRB-600 specimen under vertical loads.

This section discusses the results in the incremental test, in which each specimen was loaded for three cycles in various amplitudes. The specimen was first loaded in the vertical direction, and Figure 6 shows the force-deformation curves of the NRB-600 and LRB-600 specimen. The specimen has little visual deformation under compression loads, as shown in Figure 5, as the vertical stiffness is quite large. The force-deformation curves show that the specimen follows a predicted stiffening behavior under vertical compression loads, the obtained stiffness is listed in Table 4 and the error is within 5%. Besides, the curves, together with the parameters listed in Table 5, show that the specimen has mechanical properties approximate to the design values. The horizontal stiffness *k_h_* of the NRB-600 has the largest error, reaching 19%, since the deformation is only 50% γ_0_. The error on the horizontal stiffness is improved when the deformation increases.

**Figure 7:**
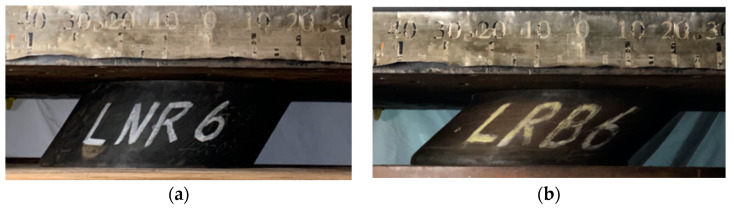
The deformation of specimens under horizontal loads: (**a**) NRB-600; (**b**) LRB-600.

**Figure 8:**
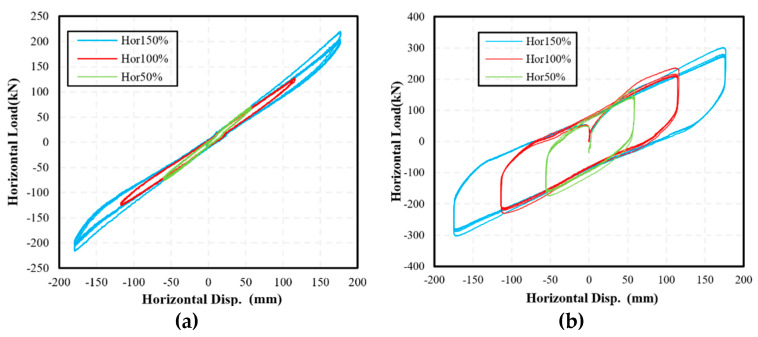
The force-deformation curves of specimens under horizontal deformation of 50%, 100% and 150% shear strain: (**a**) NRB-600; (**b**) LRB-600.

Then, the specimen was loaded in the horizontal direction, and Figure 7 shows the significant horizontal deformation of the specimen during the test. Figure 8 shows the hysteresis curves of the two specimens. Since the NRB-600 has no supplementary energy dissipation devices, it shows approximately linear behaviors under cyclic loads. Meanwhile, as the LRB-600 has a lead core, it shows significant hysteresis behaviors under the horizontal load.

As shown in Figure 6, although there is nonlinearity when the compressive deformation is small, the specimen still shows appropriately linear behavior around the rated vertical load. According to the international code ISO 22762-1, the vertical stiffness *K_v_* of the NRB and LRB can be estimated by:

(1) $$K_{v}=\frac{P_{2}-P_{1}}{Y_{2}-Y_{1}}$$

where *P*_1_ and *P*_2_ are the maximum and minimum force in the third cycle of the loading procedure, while *Y*_2_ and *Y*_1_ are the corresponding vertical deformation, respectively. Table 4 lists the horizontal stiffness *K_h_* of the NRB-600 and the post-yield stiffness *K_d_*, yield force *Q_d_* of the LRB-600. In the horizontal direction, the stiffness *K_h_* is estimated by:

(2) $$K_{h}=\frac{Q_{2}-Q_{1}}{X_{2}-X_{1}}$$

where *Q*_1_ and *Q*_2_ are the yield force at the loading amplitude, while *X*_2_ and *X*_1_ are the corresponding horizontal deformation, respectively. In addition, the LRB has the post-yield stiffness, which can be calculated by:

(3) $$K_{d}=\frac{1}{2}\left(\frac{Q_{1}-Q_{d1}}{X_{1}}-\frac{Q_{2}-Q_{d2}}{X_{2}}\right)$$

where *Q_d_*_1_ and *Q_d_*_2_ are the force values when the hysteresis curves intersect with the vertical axes. Since the specimen may exhibit different yield force at the positive and negative direction, the yield force *Q_d_* of the LRB-600 is estimated by:

(4) $$Q_{d}=\frac{1}{2}\left(Q_{d1}-Q_{d2}\right)$$

### 3.2. Results in the Cyclic Tests

**Figure 9:**
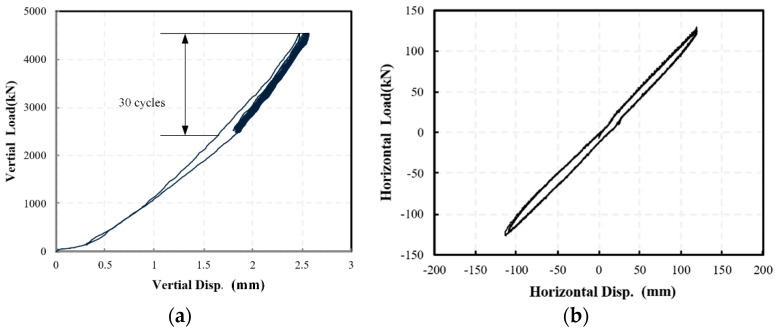
The force-deformation curves of NRB-600 specimens in (**a**) vertical and (**b**) horizontal direction.

This section discusses the results in the cyclic test, in which each specimen was loaded for 30 cycles in the same amplitudes. Figure 9 shows the force-deformation curves of the NRB-600 specimen under vertical and horizontal cyclic loads. The specimen exhibits stable mechanical behavior under the cyclic load, as the force-deformation curves in various loading cycles approximately overlap. Then, the mechanical properties of the specimen, including the vertical stiffness *K*_v_ and the horizontal stiffness *K*_h_, are estimated for the uncertainty analysis.

**Figure 10:**
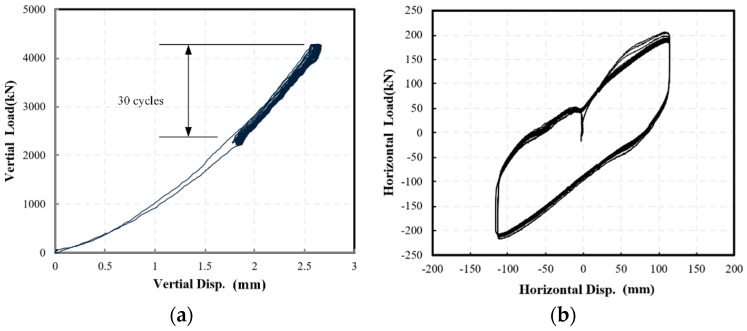
The force-deformation curves of LRB-600 specimens in the cyclic test in (**a**) vertical and (**b**) horizontal direction.

Then, the LRB-600 was also tested for 30 cycles horizontally and vertically. As shown in Figure 10, the LRB-600 specimen also showed stable mechanical behaviors. As a result, the mechanical properties of the specimen, including the vertical stiffness *K_v_*, the horizontal stiffness *K_h_*, the post-yield stiffness *K_d_* and yield force *Q_d_*, are estimated for the uncertainty analysis.

**Figure 11:**
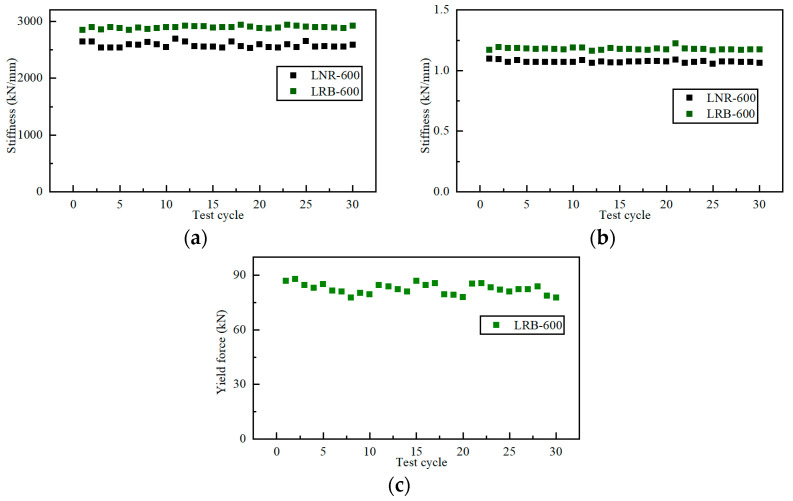
The mechanical properties of the specimens in the cyclic test: (**a**) vertical stiffness; (**b**) horizontal stiffness; (**c**) yield force.

Figure 11 shows the obtained mechanical properties of the two specimens. In the vertical direction, both the NRB and LRB shows a vertical stiffness with no obvious trend with the cycle number, which means the discontinuous repeated load has no significant influence on the vertical stiffness. In addition, the LRB shows smaller variation in the vertical stiffness than the NRB, potentially contributing from the lead core.

In the horizontal direction, as shown in Figure 11b,c, the two specimens also show stable mechanical behaviors, which are independent from the cycle number. Note that the horizontal stiffness of the LRB specimen is the post-yield one, which is much smaller than the initial one.

In summary, the cyclic test shows that the NRB and LRB specimen have reliable mechanical properties, with considerable variations, in both the horizontal and vertical direction. Particularly, the repeated load has little effect on the mechanical behaviors, the measured values are independent from the cycle number.

## 4. Discussion

The test results show that the mechanical properties of the rubber bearing specimens vary in a certain range in the cyclic test. In other researches, the mechanical properties of rubber bearings are supposed to follow a normal distribution. To find the actual distribution of mechanical properties of rubber bearings, four kinds of distribution functions are introduced, namely the generalized extreme value distribution (GEV), the normal distribution (NORM), the lognormal distribution (LOGN) and the extreme Type-Ⅰ distribution (EV). Table 6 lists the candidate parameters and expressions of the four distributions.

**Figure 12:**
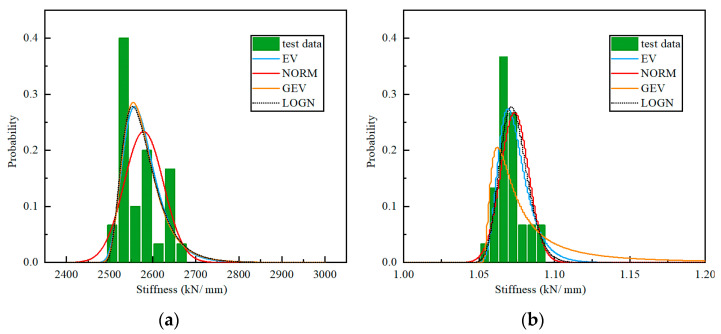
Probabilistic distribution of the NRB-600 mechanical properties: (**a**) vertical stiffness; (**b**) horizontal stiffness.

With the four probabilistic distribution functions, the parameters are derived using the built-in fitting program in the Scipy package of Python. Figure 12 shows the probabilistic distribution of the vertical and horizontal stiffness of the NRB-600 specimen. The test distribution of the vertical horizontal stiffness offset from the NORM distribution significantly. Meanwhile, the test distribution fits the LOGN and GEV distribution well, therefore, the mechanical properties of the NRB bearing can be described using the GEV and LOGN distribution, as listed in Table 7.

**Figure 13:**
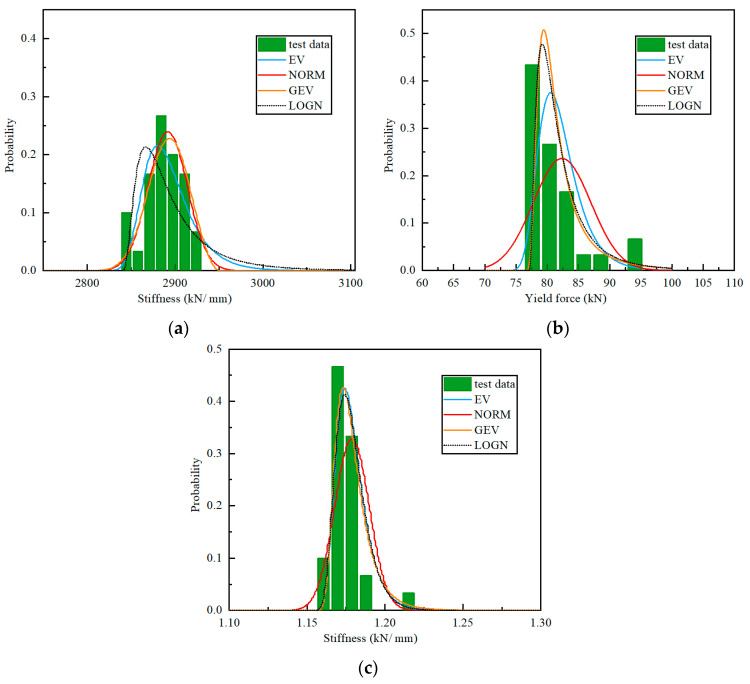
Probabilistic distribution of the LRB-600 mechanical properties: (**a**) vertical stiffness; (**b**) yield force; (**c**) post-yield stiffness.

The LRB specimen has three key mechanical properties, as shown in Figure 13. Similar to those of the NRB specimen, the mechanical properties of the LRB are also away from the NORM distribution significantly. Meanwhile, the LOGN distribution, as well as the GEV distribution, both match the test distribution well. Particularly, the LOGN distribution is easy to use as it has only two parameters. Meanwhile, the GEV has a better fitting on the vertical stiffness of the LRB than the LOGN. Table 8 lists the results of the Kolmogorov-Smirnov test of the test data under the four probabilistic density function. Therefore, the NORM and EV distribution are not recommended for the LRB.

Since the GEV and LOGN distribution show excellent match on the probabilistic distribution of all the concerned parameters, it is suggested to use the GEV or LOGN distribution. Table 9 lists the parameters of the GEV and LOGN distribution, fitted with the test results, where *μ* is the average value, *σ* is the standard deviation, ξ is the coefficient in the GEV, and COV is the coefficient of variation. The averaged values (*μ*) approximate to the design values listed in Table 1. The COVs in the stiffness properties, including the horizontal and vertical ones, are within 2%, so the variation in the stiffness is quite small. The variation in the yield force is slightly larger, reaching 5.6%.

The test results show that the mechanical properties of the NRB and LRB bearings can be described by the GEV or LOGN distribution. As a comparison, the test by Zhang et al. [25] shows that the ultimate shear deformation follows the NORM distribution, with a COV of 0.17. Besides, the test by Zhang and Li [24] also show that the yield force follows the LOGN distribution with a COV of 0.01. In this test, the COV of the mechanical properties are around 0.01 to 0.05, approximating to the results by Zhang and Li [24]. Besides, the test COV is much smaller than the assumed values, which are around 0.1–0.2 in several studies.

In this test, a certain specimen is loaded for many times to obtain the probabilistic distribution of mechanical properties. The uncertainty primarily contributes from the test error and the changes in the specimen due to repeated loads. Therefore, as this test excludes the uncertainty due to the manufacture procedure, the COV is relatively small. In addition, an isolator may be subjected to several small or moderate earthquakes during its service life. Besides, the isolator may also experience strong winds. As a result, the obtained probabilistic distribution in this study is recommended for the estimation of mechanical properties of an isolator during their service life.

## 5. Conclusions

In this study, two rubber bearing specimens were designed and fabricated. The basic mechanical properties of the bearings, including horizontal and vertical stiffness as well as yield force and its distribution function, were further tested by cyclic vertical and horizontal load tests. Based on the test results and the corresponding analysis, the following conclusions are drawn.

(1) The random variables (mechanical properties) of the specimens were statistically analyzed, and a comparison by fitting four different distributions showed that the mechanical properties, including horizontal and vertical stiffness, of typical bearing specimens NRB-600 or LRB-600 followed a lognormal or general extreme distribution.

(2) Statistical analysis shows that the coefficient of variation and standard deviation of the LRB-600 yield force are large, which is mainly because the yield force of the lead core in the bearing is not only influenced by the mechanical properties and dimensional characteristics of the lead core, but also by the interaction between the steel plate and the lead core, and there are more uncertainty factors. Therefore, special attention needs to be paid to the influence of the variability of the yield force of LRB on the seismic performance of the isolated structure in the design of the isolated structure.

(3) Statistical analysis shows that the coefficient of variation and standard deviation of the LRB-600 yield force are large because the yield force of the lead core in the bearing is often affected by the shear deformation of the inter-layer steel plate under the horizontal load. Therefore, the special attention needs to be paid to the influence of the variability of the LRB yield force on the seismic performance of the isolated structure in the design of the isolated structure.

## Figures and Tables

**Table 1:** Mechanical properties of the rubber bearings.

Specimen	$K_{v}$ (kN/mm)	$K_{h}$ (kN/mm)	$K_{d}$ (kN/mm)	$Q_{d}$ (kN)	$G_{s}$ (Mpa)	$T_{r}$ (mm)	$t_{r}$ (mm)
NRB-600	2700	1.00	—	—	0.392	120	5
LRB-600	2500	—	1.20	80	0.392	118	5

**Table 2:** Measured geometry of the specimens.

Item	Specimen	Measure Point (mm)				Average
Diameter		*D*_1_	*D*_2_	*d*_1_	*d*_2_	
	NRB-600	599	596	—	—	—
	LRB-600	598	599	121	121	—
Height		*a*	*b*	*c*	*d*	
	NRB-600	250	248	249	249	249
	LRB-600	220	219	219	219	219

**Table 3:** Test scenarios of the specimen.

No.	Specimen	Loading Style	Compression Load (MPa)	Horizontal Displacement (mm)	Loading Curve	Cycles
1	NRB-600	Axial	12 ± 30%	—	Ramp	4
2	NRB-600	Shear	12	±60, ±120, ±180	Sine	4
3	LRB-600	Axial	12 ± 30%	—	Ramp	4
4	LRB-600	Shear	12	±59, ±118, ±177	Sine	4
5	NRB-600	Axial cycle	12 ± 30%	—	Ramp	30
6	NRB-600	Shear cycles	12	±120	Sine	30
7	LRB-600	Axial cycle	12 ± 30%	—	Ramp	30
8	LRB-600	Shear cycles	12	±118	Sine	30

**Table 4:** Mechanical properties of the specimen obtained in the vertical compression tests.

Scenario	Force Max (kN)	Force Min (kN)	Deformation Max (mm)	Deformation Min (mm)	$K_{v}\;\mathrm{Test}$ (kN/mm)	$K_{v}\;\mathrm{Design}$ (kN/mm)	Error (%)
1	4279.86	2258.31	2.59	1.83	2640.82	2700	−2.19
3	4271.72	2262.82	2.65	1.88	2585.46	2500	3.42

**Table 5:** Mechanical properties of the specimen obtained in the horizontal shear tests.

Scenario	Specimen	Compression (MPa)	Shear Strain	$K_{h}\;\mathrm{Test}$ (kN/mm)	$K_{d}\;\mathrm{Test}$ (kN/mm)	$Q_{d}$ (kN)
2	NRB-600	12	±50%	1.19	—	—
			±100%	1.08	—	—
			±150%	1.00	—	—
4	LRB-600	12	±50%	—	1.23	74.69
			±100%	—	1.20	81.65
			±150%	—	1.14	87.21

**Table 6:** Parameters of the four probabilistic distribution function.

Distribution	Parameters	Probabilistic Density Function
GEV	*ξ*, $\mu_{1}$, $\sigma_{1}$	$f\left(x丨\xi ,\mu ,\sigma \right)=\sigma^{-1}\exp\left(1+\xi {\left(\frac{x-\mu }{\sigma }\right)}^{\xi^{-1}}\right){\left(1+\xi \left(\frac{x-\mu }{\sigma }\right)\right)}^{-1-\xi^{-1}}$
NORM	$\mu_{2},\sigma_{2}$	$f\left(x丨\mu ,\sigma \right)=\frac{1}{\sigma \sqrt{2\pi }}\exp\left(\frac{-{\left(x-\mu \right)}^{2}}{2\sigma^{2}}\right)$
LOGN	$\mu_{3},\sigma_{3}$	$f\left(x丨\mu ,\sigma \right)=\frac{1}{x\sigma \sqrt{2\pi }}\exp\left(\frac{-{\left(\ln x-\mu \right)}^{2}}{2\sigma^{2}}\right)$
EV	$\mu_{4},\sigma_{4}$	$f\left(x丨\mu ,\sigma \right)=\sigma^{-1}\exp\left(\frac{x-\mu }{\sigma }\right)\exp\left(-\exp\left(\frac{x-\mu }{\sigma }\right)\right)$

**Table 7:** Statistic and *p*-value in the Kolmogorov-Smirnov test of the NRB specimen.

Parameter	$K_{v}$		$K_{h}$	
	Statistic	*p*-Value	Statistic	*p*-Value
GEV	0.112	0.808	0.239	0.054
NORM	1.000	0.000	0.854	0.000
EV	1.000	0.000	0.706	0.000
LOGN	0.112	0.810	0.116	0.776

**Table 8:** Statistic and *p*-value in the Kolmogorov-Smirnov test of the LRB specimen.

Parameter	$K_{v}$		$K_{d}$		$Q_{d}$	
	Statistic	*p*-Value	Statistic	*p*-Value	Statistic	*p*-Value
GEV	0.127	0.676	0.097	0.913	0.082	0.978
NORM	1.000	0.000	0.877	0.000	1.000	0.000
EV	1.000	0.000	0.731	0.000	1.000	0.000
LOGN	0.218	0.099	0.096	0.922	0.078	0.987

**Table 9:** Parameters of the probabilistic distribution of the mechanical behaviors.

Parameter	NRB-600		LRB-600		
	$K_{v}$	$K_{h}$	$K_{v}$	$K_{d}$	$Q_{d}$
μ (kN/mm)	2579.2	1.07	2891.7	1.18	82.3
σ (kN/mm)	45.93	0.01	22.16	0.01	4.63
ξ (for GEV)	−0.062	0.147	2.63	−0.0240	−0.37
COV (%)	1.8	0.86	0.77	0.93	5.6

## Data Availability

Data are available upon requestion.

## References

[1] Zhou F., Tan P. (2018). Recent progress and application on seismic isolation energy dissipation and control for structures in China. Earthq. Eng. Eng. Vib..

[2] Ren X., Lu W., Zhu Y., He Y., Li T. (2020). Compressive behavior of low shape factor lead-rubber bearings: Full-scale testing and numerical modeling. Eng. Struct..

[3] Wu D., Tesfamariam S., Xiong Y. (2022). FRP-laminated Rubber Isolator: Theoretical Study and Shake Table Test on Isolated Building. J. Earthq. Eng..

[4] Yuan Y., Wei W., Tan P., Igarashi A., Zhu H., Iemura H., Aoki T. (2016). A rate-dependent constitutive model of high damping rubber bearings: Modeling and experimental verification. Earthq. Eng. Struct. Dyn..

[5] Wu D., Xiong Y., Yang Z. (2022). Numerical and experimental study of mechanical behaviors of the steel-confined rubber bearing. Constr. Build. Mater..

[6] Cho C.B., Kim Y.J., Chin W.J., Lee J.Y. (2020). Comparing rubber bearings and eradi-quake system for seismic isolation of bridges. Materials.

[7] Chaudhuri A., Chakraborty S. (2006). Reliability of linear structures with parameter uncertainty under non-stationary earthquake. Struct. Saf..

[8] Gupta S., Manohar C.S. (2006). Reliability analysis of randomly vibrating structures with parameter uncertainties. J. Sound Vib..

[9] Wu Q., Yan H., Zhu H., Ding L. (2020). Probabilistic performance-based assessment for critical separation distance of adjacent buildings: Theoretical analysis. J. Perform. Constr. Facil..

[10] Matsuzaki H. (2022). Time-dependent seismic reliability of isolated bridges considering ageing deterioration of lead rubber bearings. Struct. Infrastruct. Eng..

[11] Ma Y., Li Y., Zhao G., Zhou F. (2019). Experimental research on the time-varying law of performance for natural rubber laminated bearings subjected to seawater dry-wet cycles. Eng. Struct..

[12] Han R., Li Y., van de Lindt J. (2014). Seismic risk of base isolated non-ductile reinforced concrete buildings considering uncertainties and mainshock–aftershock sequences. Struct. Saf..

[13] Mishra S.K., Chakraborty S. (2013). Performance of a Base-Isolated Building with Sys-tem Parameter Uncertainty Subjected to a Stochastic Earthquake. Int. J. Acoust. Vib..

[14] Fan J., Long X., Zhang Y. (2015). Optimum design of lead-rubber bearing system with uncertainty parameters. Struct. Eng. Mech. Int. J..

[15] Nassar M., Guizani L., Nollet M.J., Tahan A. (2022). Effects of temperature, analysis and modelling uncertainties on the reliability of base-isolated bridges in Eastern Canada. Structures.

[16] Saha S.K., Matsagar V., Chakraborty S. (2016). Uncertainty quantification and seismic fragility of base-isolated liquid storage tanks using response surface models. Probabilistic Eng. Mech..

[17] Alhan C., Hisman K. (2016). Seismic isolation performance sensitivity to potential deviations from design values. Smart Struct. Syst..

[18] Li H., Xu Z., Gomez D., Gai P., Wang F., Dyke S.J. (2022). A modified fractional-order derivative zener model for rubber-like devices for structural control. J. Eng. Mech..

[19] Politopoulos I., Pham H.K. (2009). Sensitivity of seismically isolated structures. Earthq. Eng. Struct. Dyn..

[20] Scozzese F., Dall’Asta A., Tubaldi E. (2019). Seismic risk sensitivity of structures equipped with anti-seismic devices with uncertain properties. Struct. Saf..

[21] Gazi H., Alhan C. (2018). Probabilistic sensitivity of base-isolated buildings to uncertainties. Smart Struct. Syst..

[22] Zhou W., Wang C., Fan P., Kuang Y., Dong Z. (2022). The Sealing Effect Improvement Prediction of Flat Rubber Ring in Roller Bit Based on Yeoh_Revised Model. Materials.

[23] Xu X., Zhang Z., Hu Y., Wang X. (2020). Bearing strength of crumb rubber concrete under partial area loading. Materials.

[24] Zhang R., Li A. (2022). Probability distribution characteristics and statistical parameters of the horizontal stiffness of rubber isolation bearings. J. Earthq. Eng..

[25] Zhang F., Gu M., Lu F., Dong Y., Pei M. (2013). Probabilistic and statistical analysis of rubber bearing’s limit horizontal displacement. Eur. J. Environ. Civ. Eng..

[26] Kumar M., Whittaker A.S., Kennedy R.P., Johnson J.J., Kammerer A. (2017). Seismic probabilistic risk assessment for seismically isolated safety-related nuclear facilities. Nucl. Eng. Des..

[27] Ahmadipour M., Alam M.S. (2017). Sensitivity analysis on mechanical characteristics of lead-core steel-reinforced elastomeric bearings under cyclic loading. Eng. Struct..

[28] Faber M.H. (2005). On the Treatment of Uncertainties and Probabilities in Engineering Decision Analysis. J. Offshore Mech. Arct. Eng..

[29] Der Kiureghian A., Ditlevsen O. (2009). Aleatory or epistemic? Does it matter. Struct. Saf..

[30] Zhai C., Wen W., Ji D., Li S. (2015). The influences of aftershocks on the constant damage inelastic displacement ratio. Soil Dyn. Earthq. Eng..

[31] Basim M.C., Pourreza F., Mousazadeh M., Hamed A.A. (2022). The effects of modeling uncertainties on the residual drift of steel structures under mainshock-aftershock sequences. Structures.

[32] (2018). Elastomeric Seismic-Protection Isolators-Part 1: Test Methods.

